# Women’s experiences with breast cancer during diagnosis and therapy, Wolaita, Ethiopia: a qualitative study

**DOI:** 10.1186/s12905-024-03016-z

**Published:** 2024-03-13

**Authors:** Beimnet Desalegn Kedida, Mihiretu Mohammed Mukacho, Mihiretu Alemayehu, Serawit Samuiel, Sintayehu Kussa, Yordanos Sisay, Desta Markos, Worku Mimani

**Affiliations:** 1 School of Public Health, Collage of Health Science and Medicine, Wolaita Sodo University, P. O. Box: 138, Wolaita Sodo, Ethiopia; 2 Wolaita Sodo University Comprehensive Specialty Hospital, Wolaita Sodo, Ethiopia

**Keywords:** Breast cancer, Women’s experiences, Qualitative study, Wolaita, Ethiopia

## Abstract

**Background:**

Breast cancer is the most common cancer in women and the most frequent cancer worldwide. After being diagnosed with breast cancer, women experience unexpected and stressful events. In Ethiopia, specifically in the study area, the experiences of women with breast cancer, the challenges they face during treatment and follow-up have not been thoroughly investigated.

**Objective:**

This qualitative study explores the experiences of women diagnosed with breast cancer and undergoing therapy at a University-based hospital in Ethiopia.

**Methods:**

A qualitative research design was used, to explore the experiences of women diagnosed with breast cancer and undergoing therapy. A purposively selected sample of ten women who had been diagnosed with breast cancer and were receiving therapy was recruited. Recruitment was conducted from August 1 to September 30, 2022. Semi-structured face-to-face interviews were conducted to collect data on their experiences. The interviews were transcribed verbatim, and a thematic analysis approach was employed utilizing open coding. The coded data were then analysed to reveal important insights and understandings about the participants’ experiences with breast cancer during the diagnosis and therapy journey.

**Result:**

The thematic analysis of the data revealed four prominent themes: women’s mixed emotions, characterized by a sense of high threat and hope upon receiving breast cancer diagnosis results; dealing with the changes, both physical and psychological, that the cancer and its treatment bring about in women’s bodies and emotional well-being; dealing with the challenges associated with accessing therapy, including unfavourable hospital conditions and financial hardship; and experiencing care and supports from health care providers, family and friends, and faith-based communities.

**Conclusion:**

These findings underscore the importance of providing comprehensive support and care for women with breast cancer. Enhancing the hospital environment, addressing resource shortages, and prioritising patient well-being are crucial steps towards improving the experiences of breast cancer patients in the study area.

## Introduction

Breast cancer is the most common cancer in women and the most common cancer worldwide. The incidence of breast cancer is higher in industrialised countries, whereas the proportional fatality rate is highest in developing countries [1].

Evidence shows that breast cancer is currently the most frequent malignancy in women in developing countries. This represents a significant departure from past decades when cervical cancer was the most often diagnosed cancer [2]. According to the World Health Organization (WHO), as of the end of 2020, approximately 2.3 million women will be diagnosed with breast cancer, with 685,000 deaths worldwide. In the previous five years, 7.8 million women had been diagnosed with breast cancer, which making it the most frequent in the world. It claimed the lives of more than 74,072 people in Africa, with 168,690 new cases reported [3].

Breast cancer can have a number of effects on a woman’s daily life, depending on the stage of the disease and the sort of treatment she receives [4–7]. Currently, numerous cancer treatments and medications are available, such as surgery and radiation therapy, to treat a specific tumour or area of the body, and others are drug treatments, such as chemotherapy, immunotherapy, or targeted therapy. Cancer therapies have a variety of side effects. Adverse effects might vary from person to person, as well as between medicines and types of treatment.

Obtaining effective cancer diagnosis and treatment is a significant difficulty in poor countries. This has a substantial impact on women’s quality of life [8]. Breast cancer patients in Ethiopia had a lower quality of life than others, and they were dissatisfied with emotional support and care [9]. Previously, access to public oncology treatment in Ethiopia was limited to Black Lion Specialised Hospital [10]. However, recognizing the urgent need for expanded cancer care, the Ethiopian Minister of Health has taken proactive measures. A significant development in this regard is the establishment of Ethiopia’s second radiology center at Jima University Hospital. Additionally, specialised hospitals across the country now offer screening and follow-up services for cancer patients. Wolaita Sodo Comprehensive Specialty Hospital also offers cancer screening, treatment, and follow-up services except radiation treatment for cancer patients, which has thus far been available only at the Black Lion and Jimma Specialised Hospital in Ethiopia. By establishing more specialised facilities and broadening the range of services available, the goal is to enhance the quality of cancer care and improve outcomes for patients throughout Ethiopia.

Breast cancer and its treatment have a negative impact on the physical, psychological, and spiritual aspects of breast cancer survivors [11, 12]. Evidence suggests that women with breast cancer have varying reactions to the disease, and these reactions influence their coping strategies and disease treatment [13]. Physical, psychological, and social concerns are also among the early and late side effects of breast cancer and its treatments. Breast cancer has a higher impact on patients’ self-esteem and quality of life [8, 9, 14].

Patients with breast cancer have physical, emotional, and social changes. As a result, breast cancer is commonly perceived as a threat to a woman’s sense of femininity as well as her life [15]. Breast removal during cancer treatment has been shown to have a major impact on a patient’s perception of femininity [16, 17]. Breast cancer patients are frustrated by body changes, avoid revealing their physical selves, reduce social status, and mourn the loss of their former attractive selves [18]. After taking chemotherapy, a patient may feel weakness, bodily pain, various types of neuropathy and tingling in their extremities, blood clots, short-term memory loss, lingering cough, skin changes, and a metallic taste in their mouth [19].

Cancer is quickly becoming Ethiopia’s biggest public health threat. Breast cancer is now receiving attention in terms of research [10]. Several studies on breast cancer in Ethiopia have focused on quality of life, late diagnosis, breast cancer survival, and quality of life with chemotherapy [8]. Nevertheless, in Ethiopia, rather than opening screening and follow-up centres, particularly in the study area, the experiences of women with breast cancer, the obstacles they confront throughout treatment and follow-up, and the forms of care available to aid them have not been adequately examined. As a result, the information in this qualitative study on women’s life experiences with breast cancer will help to understand the challenges they confront while undergoing breast cancer treatment and to improve the service given by health institutions.

Despite extensive research efforts on various aspects of breast cancer, such as quality of life, late diagnosis rates, survival rates, and the impact of chemotherapy, there remains a lack of comprehensive understanding regarding the obstacles faced by patients during diagnosis, treatment, and follow-up, as well as the available forms of care to support them. In particular, the experiences and challenges of breast cancer patients in newly established oncology centers in Ethiopia are largely unknown. This knowledge gap highlights the need for qualitative studies that delve into the life experiences of women with breast cancer during diagnosis and treatment. Therefore, the aim of this study was to explore the experiences of women diagnosed with breast cancer and undergoing therapy at a University-based hospital in Ethiopia. This knowledge will facilitate the development and implementation of more targeted interventions and services that address the specific challenges faced by breast cancer patients throughout their journey, ultimately leading to improved outcomes and quality of life for these individuals.

## Materials and methods

### Study design and setting

A qualitative study was conducted to explore the experiences of women diagnosed with breast cancer and undergoing therapy. The research took place at Wolaita Sodo University Comprehensive Specialised Hospital located in Wolaita Sodo, South Ethiopia. This the comprehensive healthcare facility consisting of nine departments, including an oncology clinic. The hospital serves a population of approximately 3–5 million people from the Wolaita zone and surrounding areas. Currently, the hospital caters to over 500 patients daily, with the oncology department offering services to a significant number of patients. The department comprises three wards and is staffed by one oncologist, four medical doctors, and nine nurses. According to the 2021 report, the oncology ward treated around 800 cancer patients. Services provided by the oncology department include oncology outpatient care, inpatient services, and pharmacy services. The oncology inpatient unit has a capacity of 12 beds. The study was conducted from August 1 to September 30, 2022, within this healthcare setting.

### Participants recruitment

Purposive sampling was employed to select participants for the study. The process involved deliberate and intentional selection based on specific criteria relevant to the research objectives. The following steps were followed in undertaking the purposive sampling: Identification of target population: - The target population was defined as women diagnosed with breast cancer and receiving follow-up care at Wolaita Sodo University Comprehensive Specialised Hospital in 2022. Clear inclusion criteria were established to guide participant selection. These criteria included women who had been diagnosed with breast cancer at least six months prior, who were currently undergoing any form of therapy, and who were 18 years of age or older. Exclusion criteria were also defined to ensure that the sample represented the desired population. Participants with mental health illnesses, severe comorbidities, or who were unwilling or declined to participate were excluded.

The principal investigator collaborated with the oncology clinic staff to review the clinical profiles of potential participants. This collaboration helped identify women who met the inclusion criteria and were suitable candidates for the study. The selection of participants was purposeful and based on the identified criteria. The aim of this study was to ensure a diverse range of experiences and perspectives related to the diagnosis and therapy of breast cancer. Sampling continued until data saturation was reached. In this case, the researchers determined that data saturation was achieved after interviewing a total of 10 participants.

### Data collection procedure

To gain a deeper understanding of women’s experiences with breast cancer, in-depth interviews (IDIs) were conducted. A topic guide was developed using relevant literature as a reference. In-depth interviews were carried out with breast cancer patient, utilizing a team of two experienced researchers who possess a strong background in qualitative data collection. The principal investigator (PI) played a crucial role as both a data collector and facilitator, ensuring the smooth progress of the interviews. To maintain objectivity and minimize potential biases, the PI had no prior connections or relationships with the research participants. The PI carefully selected a conducive and comfortable environment for the interviews, such as a private consultation room at the hospital or a quiet and confidential space at a participant house. These locations aimed to create a safe and supportive atmosphere that encouraged participants to openly share their experiences and perspectives. The participants were asked questions and prompted to share their feelings about the diagnosis results, how they coped with the situation, and their experiences throughout the therapy and follow-up process at the cancer clinic (Table 1). During the interviews, participant statements prompted further exploration and clarification of their experiences. In addition to interviews, memos were collected, offering valuable insights and enriching the analysis of the research topic.

**Table 1:** Interview guide

S.no	Interview guide
1.	How did you feel emotionally upon receiving your breast cancer diagnosis?
2.	Can you describe the physical and psychological changes you experienced as a result of the cancer and its treatment?
3.	What challenges did you face in accessing therapy for your breast cancer, such as hospital conditions?
4.	How have healthcare providers, family, and friends supported you during your breast cancer journey?
5.	Can you share any moments or experiences that have given you hope or helped you cope with the challenges of breast cancer?
6.	How do you manage the day-to-day emotional and psychological impact of breast cancer?
7.	What coping strategies or resources have been particularly helpful to you in dealing with the challenges of breast cancer?
8.	Have you encountered any difficulties in communicating your needs and concerns to healthcare providers?
9.	How has the breast cancer diagnosis and treatment affected your relationships with family, friends, and others in your support network?

Simultaneous data collection and analysis took place during the study. In-depth interviews (IDIs) were conducted in Amharic, the participant’s native language. To ensure thorough data collection, multiple visits and interviews were carried out with each participant until data saturation was achieved. New concepts and insights were incorporated, and main themes were compared across interviews to identify common patterns and variations. To preserve the context, the IDI audio recordings were transcribed verbatim, and the sound files were encrypted and securely stored. The initial transcriptions were done in Amharic and to ensure accurate analysis, the transcriptions were later translated into English by professional and experienced translators, while upholding the integrity and fidelity of the data.

### Data analysis

Data obtained from the interviews were analysed using thematic analysis [20, 21]. The interviews conducted in this study were transcribed, and the audio recordings were merged with field notes taken during the sessions. Researchers immerse themselves in the data by reading and re-reading the transcripts to familiarize themselves with the data. Then the data underwent an initial open coding process using Open Code software version 4.02, whereby each line of the data was coded. This involved identifying and assigning codes to segments of data that are relevant to the objectives of the study. This process involved labelling or tagging specific ideas, concepts, or patterns within the data. The codes are then reviewed and grouped together to identify broader patterns or themes that emerge across the dataset. The resulting list of codes and their corresponding descriptions were summarized into a codebook (Table 2). This involves identifying commonalities, differences, and variations within the data. Researchers review and refine the identified themes to ensuring they accurately capture the essence of the data. Finally, each theme is defined and described, accompanied by illustrative quotes from the data to support its interpretation. The findings are synthesized and presented in a narrative form, describing the themes, their interrelationships, and their significance in answering the research question or addressing the research objectives.

**Table 2:** The themes and sub-themes Emerged from women’s living with breast cancer at WSUCSH, Wolaita, South Ethiopia, 2022

Themes	Sub-themes
Women’s mixed emotions	High threat Hope
Dealing with changes	Physical changes Psychological changes
Dealing the challenges of getting therapy	Unfavourable hospital situation Financial hardship
Care and Support	Health care provides support Family and friends support Faith-Based Support

### Trustworthiness of the study

In this qualitative study, the researcher implemented various strategies to uphold trustworthiness [22, 23]. First, credibility was enhanced through prolonged engagement with participants, allowing for an in-depth understanding of their experiences. Multiple visits and interviews were conducted to comprehensively explore their perspectives. Triangulation, which was achieved by merging audio recordings with field notes, was used to validate and corroborate the collected data.

Second, transferability was ensured by providing a detailed description of the research setting, specifically the hospital and its oncology department. The inclusion and exclusion criteria for participant selection were clearly outlined to ensure that the sample represented the target population. This information enables readers to assess the applicability of the findings to other contexts or populations.

Third, dependability was fostered through meticulous documentation of the research procedures, data collection, and analysis methods. This transparency allows for potential replication or verification by other researchers. The use of a purposive sampling method and well-defined participant selection criteria further contribute to the dependability of the study’s findings.

Moreover, confirmability was promoted by engaging in reflexivity, critically reflecting on the researcher’s biases and assumptions throughout the research process. This self-awareness minimized the potential influence of preconceptions on data interpretation. Maintenance of an audit trail, documenting decision-making processes, ensured transparency and objectivity.

Finally, member checking was employed as a strategy to enhance trustworthiness. Participants were given the opportunity to review and provide feedback on the findings, ensuring that their perspectives were accurately represented in the final analysis.

## Results

Ten women diagnosed with breast cancer participated in an in-depth interview for this study. The women’s ages ranged from 20 to 52 years. In terms of educational background, the majority of the participants had completed primary school or higher. Among the participants, seven women were married, seven were Protestant religious followers, and five were involved in merchant occupations. Eight of the women resided in urban areas, while the remaining two were from rural areas. The duration of their breast cancer diagnosis ranged from 15 to 26 months. More details can be found in Table 3.

**Table 3:** Background characteristics of the study participants, Wolaita, south Ethiopia, 2022 (*n* = 10)

ID	Age	Educational status	Marital status	Religion	Occupation	Residence	Duration of diagnosis
IDIP01	52	Primary school	Married	Protestant	House wife	Rural	2 year
IDIP02	30	Diploma	Married	Protestant	Merchant	Urban	16 months
IDIP03	38	No formal education	Married	Orthodox	Housewife	Urban	12 months
IDIP04	20	Secondary school	Unmarried	Protestant	Merchant	Urban	18 months
IDIP05	35	Secondary school	Married	Protestant	Merchant	Urban	2year
IDIP06	35	Primary school	Married	Protestant	House wife	Ruler	16 months
IDIP07	40	No formal education	Married	Protestant	House wife	Urban	14 months
IDIP08	25	Secondary school	Unmarried	Orthodox	Student	Urban	26 months
IDIP09	45	Primary school	Unmarried	Orthodox	Merchant	Urban	18 months
IDIP10	24	Diploma	Married	Protestant	Employed	Urban	15 months

The analysis revealed the identification of 9 subthemes, which were further condensed into 4 main themes. The themes extracted from the data were as follows: Women’s mixed emotions, dealing with changes, dealing the challenges of getting therapy, and receiving care and support. The specific themes and subthemes can be found in Table 2.

### Theme 1: Mixed emotions

The participants described experiencing a range of emotions upon receiving their breast cancer diagnosis results. These emotions were divided into two subthemes: high threat and hope. High-threat emotions included feelings of shock, fear, and worry, reflecting the initial distress and anxiety associated with the diagnostic results. On the other hand, the presence of hope indicated the participants’ resilience and the potential for positive emotional experiences throughout their breast cancer journey. This mix of negative and positive emotions showcases the complex psychological response that individuals experience when receiving breast cancer diagnosis results.

#### High threat

The women described experiencing intense emotions of shock, fear, and worry upon receiving their breast cancer diagnosis results. The news came as a significant threat, disrupting their sense of well-being and causing distress. The sudden and unexpected nature of the diagnosis contributed to feelings of shock, leaving them in a state of disbelief and emotional turmoil. They also expressed fear about the implications of the diagnosis, including concerns about their health, treatment options, and prognosis. Worry about the impact on their lives and the future further intensified their emotional distress.

An uncomfortable condition of mental disquiet, tension, and apprehension, caused by the results of the diagnosis and concern about the disease’s prognosis over the possibility of anticipated sequence. Of the study participants, almost all reported that they had such feeling when they first heard about the result of their cancer diagnosis.

When I heard the word “cancer” I was fainted and became unconscious for minutes… because cancer (breast cancer) is a dangerous disease that may lead to loss of the breast and the life of women… (P6,28 years old women).

The first time I came to this hospital… He (Doctor) had examined me and when told that it was cancer… I started to cry… because I can’t eliminate it, … After all, I’d go anxious thinking about my life… (P8, 25 years old woman).

The participants expressed significant concerns, including fear of losing their breasts, apprehension about the severity of the disease, and doubts about the potential for improvement. They perceived these factors as high threats, which fuelled their emotional distress. In addition, the participants repeatedly mentioned that their families shared their worries about the uncertain prognosis of the disease, further contributing to their personal anxiety.

because we had seen individuals who amputated their hands and feet there (in hospital) … my family was very shocked and my husband had cried thinking that the same thing would happen to me… breast is a big thing and the situation was very difficult and made me anxious. (P2, 30 years old woman)

#### Hope

Alongside the negative emotions, participants also reported feelings of hope. Hope in the context of women diagnosed with breast cancer refers to their optimistic outlook and belief that something positive will happen, relying on their faith in God for a solution. Despite being fully aware of their illness, these women maintain a positive attitude towards their condition and their future life. This condition empowers them to focus on the meaningful aspects of life and continue their pursuit of a fulfilling existence. Their hope serves as a guiding force, allowing them to navigate through the challenges of their illness while maintaining a positive mindset.

… God will heal me from this disease, I have hope in Him and he will heal me from this disease… (P9, 45 years old woman).

I have hope in God… if I take care of myself in the future, the disease will leave me… it is getting better now, thanks to God, … I don’t feel any pain in the breast, … everything I feel around my heart has gone. (P3, 38 years old woman)

I have no bad feelings now regarding the disease. Thanks to God, I am getting better… As GOD will… I will become fully healthy, I don’t worry about today’s problems. I have hope that God will cure me (P6, 28 years old woman).

Hope played a significant role as a coping mechanism and a source of resilience for the participants, empowering them to navigate the challenges of breast cancer diagnosis. It served as a positive outlook, motivating them to actively participate in their treatment and recovery journey. Hope provided them with the strength and determination to face the difficulties head-on, fostering a sense of optimism and empowerment in their battle against breast cancer.

### Theme 2: Dealing with change

Breast cancer patients experience notable transformations because of the disease and its treatment. The participants in the study emphasised the adverse effects on their personal image and emotional well-being. This brought attention to two sub-themes: physical and psychological changes.

#### Physical change

All the women involved in the study acknowledged that their bodies had been significantly affected by the disease and the treatment they received. These changes had noticeable consequences on their physical well-being. One prominent aspect that the participants discussed was the loss of a breast.

I had breast surgery … I lost one of my breasts. … (p7,40 years old women)

The participants also shared experiences of various side effects resulting from the medication they received. These side effects included crooked nails, dizziness, palpitations, loss of appetite, and headaches. The physical discomfort caused by these side effects further affected their appetite and food preferences. The participants expressed a reduced desire for food and an aversion to its smell because of the medication’s impact on their senses. In addition to these effects, the participants mentioned changes in their skin appearance and hair loss. The treatment altered the appearance of their skin, and they experienced hair loss, leading to baldness. These physical changes added to the overall impact on their self-image and contributed to their sense of identity transformation.

Oh, my God! … Because of the drug, my nails are crooked; I have dizziness, palpitations, loss of appetite, and headaches. … I have no appetite for food I don’t like the smell of food. Fortunately, they told me it is side effects of the drugs… (p5, 35 years old woman).

Because of this treatment, the appearance of my skin has changed. My hair is lost and becoming bald. (p8, 25 years old women)

#### Psychological change

The study examined the psychological impact of the change in body appearance on patients, revealing the development of negative emotions and pessimistic thoughts about life. These psychological effects further exacerbated the challenges they faced in their daily lives. The majority of participants expressed that the treatment had a significant influence on their mental well-being.

One prominent aspect that the participants discussed was the loss of a breast, which had a profound impact on their sense of femininity. The symbolic significance of breasts for women was highlighted, with one participant expressing the meaning of breasts as a representation of life and femininity. The emotional and psychological effects of losing a breast were evident in the participants’ narratives.

I had breast surgery … I lost one of my breasts. … breast means life for women and an image for feminity, … (p7,40 years old women)

Some participants shared their discomfort and pain when discussing the loss of a breast. It was evident that this aspect of their physical transformation was emotionally distressing. They experienced a sense of hurt and chose not to disclose their breast loss to others. One participant mentioned using a breast holder to conceal the absence, but it still caused emotional pain when people inquired about their breasts.

when they ask me about my disease … I don’t tell them that I had lost breast… it hurts me, … I put something in a breast holder. …When people come with me, they ask me about my breasts. (p8, 25 years old woman)

Another participant expressed feelings of fear and avoidance when discussing their condition with others. They described a reluctance to leave the house because of the anxiety surrounding conversations about their disease. Fear of judgement and negative reactions from others contributed to a perceived sense of being disabled or different.

…but I don’t go out of the house because I have a fear of talking about my condition or disease to others… and I worry about what they would say about me when they see me. I feel I am a disabled person (p4,20 years old ladies)

These narratives highlight the profound psychological impact of the change in body appearance on the patients. Negative emotions, discomfort, fear, and a sense of being different or disabled were common themes among the participants. The psychological challenges resulting from these experiences added to the overall burden of their illness and treatment, making their lives more difficult.

### Theme 3: Dealing the challenges of getting therapy

Living with breast cancer presents significant challenges for women, as indicated by our study participants. These challenges encompassed various aspects, including the high costs associated with diagnosis and treatment, medication shortages, crowded environments at oncology clinics, and transportation difficulties during follow-up appointments. These challenges are subthemes, specifically the unfavourable clinic environment characterised by overcrowding and medication shortages, and financial constraints related to the expenses of diagnosis and treatment, as well as transportation issues.

#### The unfavourable clinic environment

The participants voiced considerable anguish regarding the inadequate availability of medication and medical supplies. They expressed frustration during follow-up appointments because of the persistent unavailability of essential items, leading to feelings of anger. Dealing with these ongoing issues left them feeling exhausted and fatigued.

Don’t raise this issue… it made me get mad during wound care. When we bring gloves, they ask for gauze. When we bring gauze, they say there is no medicine … It was a challenging time and to pass that time we used a lot of methods… now we got tired of things. (p6, 28 years old women)

There are no laboratory tests here… When we go to a private clinic, we pay for transport more than 80 birrs … and 900 birrs for the laboratory test… we finished the money by laboratory investigation alone … (p10, 25 years old woman).

According to the participants, the condition of the oncology clinic during their hospital stay was described as stressful. They reported experiencing crowdedness, noise, and a sense of suffocation due to sharing a room with other cancer patients. In addition, concerns were raised about the unclean state of the toilet facilities. The participants expressed concern that these clinical conditions in the patient ward could expose them to additional illnesses and adverse effects of medication, further adding to their concerns.

When I come for follow-up… it worries us a lot because many patients share a room and it is crowded every day … People with different types of cancer are kept in a single room. there is another option for people who have money … they can use private wing and pay for the bed. (p2, 30 years old women)

Another thing, I would like to tell you is that we are feared… the diagnosis area and the room where the patient will be admitted should be convenient and have enough space because all types of patients stay in one room… it worries a lot (p4,20 years old lady).

#### Financial hardship

Financial hardship was a major challenge faced by nearly all the participants in the study. Because of insufficient laboratory tests and medication at the oncology clinic, patients were frequently referred to private health facilities, resulting in significant expenses for services such as laboratory investigations and pharmacy purchases.

…The cost of the medications was expensive … we were bought from a private health facility, … (p8, 28 years old women).

…The biggest problem is buying medication from a private pharmacy. The current fee for medication at private pharmacies is very high, so I can’t afford for… up to twelve rounds of treatment. (p3, 38 years old woman)

The high costs of medication from private pharmacies were particularly distressing, making it difficult for participants to afford the necessary rounds of treatment. As a result, many participants experienced difficulties in covering the expenses associated with their treatment, leading to postponed follow-up appointments and financial constraints, especially for those with low incomes.

For me… It is very difficult to buy the medicine, I have no money, I have no one to help me, I have no father, mother, brother or sister, I am alone, and I administer myself alone… so I go for follow-up when I got money (P8,25 years old ladies).

Some participants resorted to sells their belongings or taking out loans to finance their treatment. The financial burden was exacerbated by the fact that some participants had no familial support and rely solely on their own resources. The impact on their livelihoods was evident, as some had to stop their businesses or experienced reduced income due to the disease’s financial implications.

We were getting money for treatment by selling our house materials and by the loan… We had finished our money at the beginning… now the remaining is… to sell the house we dwell. (P6, 28 years old women)

…Yeah, I have financial problems. I expend 4,000 to 5,000 birrs in each follow-up round, which is difficult for us(family). That is too hard for me. We have children to raise. I stopped all my businesses because of this disease. At least, I had something to do to support my family in our daily lives. However, this is an additional expense. My husband is a farmer… as you know, farming is not as productive as before due to the lack of rain. We have no additional income, which is so challenging for us. (P5, 35 years old women)

### Theme 4: Care and supports

According to the women, support played a vital role in their physical and mental well-being throughout their breast cancer journey. They emphasised that having support from healthcare professionals’, family and friends’, and faith-based communities was a crucial ingredient in maintaining their overall health. The care and support they received provided them with assistance, comfort, and encouragement, helping them cope with the emotional and practical aspects of dealing with breast cancer. They reported that this support was instrumental in promoting their resilience and contributing to their overall well-being during the challenging times they faced.

#### Healthcare provides support

According to the participants, healthcare providers, including doctors, nurses, and other medical professionals, offer specialised knowledge, guidance, and treatment options based on their expertise. They are viewed as a source of hope and strength by patients. The participants described healthcare providers as compassionate and supportive, providing emotional support, information, and guidance throughout the treatment process. Patients reported that healthcare providers addressed their concerns and reassured them, instilling confidence and reducing anxiety. For example, one participant mentioned how healthcare providers reassured her about swelling and administered an injection, demonstrating their caring nature.

This is a fourth follow-up… and when I asked them about the swelling, they (healthcare providers) reassured me… Don’t worry much they said, and gave me an injection(medication)… and they are so caring. (P5, 35 years old woman)

Other woman, shared her experience during follow-up visits with healthcare providers, stating,

When I come for follow-up, they (healthcare providers) are welcoming and examine us very well. They take the time to update us on the progress of our condition, provide advice on medication, and guide us on proper dietary habits.

This highlights the positive and comprehensive care received from healthcare providers during follow-up appointments.

#### Family and friends’ support

Participants expressed that support during diagnosis and treatment encompassed several aspects, including emotional reassurance, empathetic care, and practical assistance from their families and friends. They shared their worries and concerns about the uncertain prognosis of breast cancer with their families, who in turn provided emotional support, understanding, and shared fears and anxieties about the severity of the disease. One participant, described the shock of receiving her cancer diagnosis and how her family also experienced a profound sense of fear and concern, knowing the seriousness of the disease.

I went to the hospital alone… and after the diagnosis… they (nurses) told me that a specialist will see me… then he saw my result… he said ‘It is cancer’… then I was shocked and called and told my family… My family was very shocked too… Always they fear my death, they are very scared because they know cancer is a very serious disease. (P10, 24 years old woman)

Participants also mentioned the remarkable cooperation and sacrifice of their family members. Some families were willing to sell household materials to cover the expenses of therapy, exemplifying their dedication and support. One participant woman, expressed gratitude for the incredible support from her family, highlighting their willingness to make sacrifices for her access to necessary treatment.

My family has been incredibly supportive throughout my treatment. They have made sacrifices, like selling some of our belongings, just to ensure I can access the therapy I need. (P6, 28 years old woman)

Friends also played a significant role in providing emotional support by lending a listening ear, offering words of encouragement, and being present when needed. Their unwavering support served as pillars of strength during challenging times. Another participant highlighted the practical assistance provided by family and friends in various aspects of daily life. They helped with household chores, cooked meals, and even accompanied the participant to medical appointments, demonstrating their invaluable support.

In our rural community, our friends have been like pillars of strength for us. Whenever we needed someone to talk to, they were always there to lend a listening ear and offer words of encouragement. Their unwavering support has been our source of strength during the tough times we faced. Moreover, our family and friends have been incredibly helpful in our daily lives. They have assisted us with household chores, cooked meals for us, and even accompanied us to medical appointments. Their support has been invaluable, and we are grateful to have such caring and supportive individuals in our countryside community. (P1, 52 years old woman)

These accounts reflect the deep level of support and understanding offered by both family and friends throughout the treatment journey, demonstrating their essential role in providing emotional reassurance, empathetic care, and practical aid to individuals facing breast cancer.

#### Faith-based support

According to the accounts shared by participants, women who embrace religious or spiritual beliefs find substantial support within their faith communities. They reported that their religious circles, including religious leaders and fellow members, provide prayers, encouragement, and spiritual comfort during the difficult journey of battling breast cancer. This support has a profound impact, fostering a sense of hope and boosting their emotional well-being. The participants expressed how the involvement of their faith community uplifts their spirits and helps them navigate the challenges they face. One participant expressed the significant support she received from her church community.

My church community has been a source of immense support throughout my breast cancer journey. The prayers, encouragement, and spiritual comfort I receive from fellow members and religious leaders uplift my spirit and give me strength to face the challenges. (p5, 35 years old woman)

She emphasized the significant role played by prayers, encouragement, and spiritual comfort from fellow members and religious leaders in empowering her to confront the challenges of her breast cancer journey. In addition, another participant shared their personal experience, expressing,

I have personally incorporated the use of holy water, blessed and given to me by church leaders, to seek spiritual strength and healing. (p3, 38 years old woman)

## Discussion

Breast cancer has a profound impact on women worldwide, with diverse experiences that encompass several emotions, challenges, and triumphs. Upon receiving a diagnostic result, women embark on a challenging journey that tests their physical and emotional strength, resilience, and support systems. This study illuminates the lived experiences of breast cancer patients during diagnosis and therapy. In Ethiopia, where the healthcare system is expanding cancer care and therapy, women face additional challenges because of the novelty of the system in many healthcare facilities. This research seeks to provide valuable insights into the unique obstacles these women encounter, unravelling the intricacies of their journey.

The study findings revealed that women who received breast cancer diagnosis results experienced intense emotions of shock, fear, and worry, which significantly disrupted their well-being and caused emotional distress. In other studies, participants also expressed feelings of depression, instability, and frustration upon learning about their diagnosis [11, 24–26]. The unexpected nature of the diagnosis contributed to feelings of shock and disbelief, whereas concerns about health, treatment options, and prognosis further heightened their emotional distress. The abrupt diagnosis and subsequent uncertainty surrounding the disease prognosis pose a significant threat. This situation evoked shock and anxiety in response to the diagnostic results. A study conducted in Ghana found that women experienced emotional reactions such as despair, worry, and anxiety due to various factors, including the financial burden of treatment and the fear of mortality [26, 27]. Fear of losing their breasts, worries about the severity of the disease, and doubts about improvement were significant concerns that added to their emotional distress. Furthermore, the participants’ families shared these worries, amplifying their anxiety. Another study revealed that women who were living with breast cancer described their experience as a profound loss in life, resulting in a loss of confidence and living in constant fear [15, 16, 28]. The impact of the disease on their lives was profound, leading to a sense of deprivation and a shift in their overall outlook. The emotional toll of living with breast cancer was evident as they grappled with the challenges and uncertainties associated with the illness. These women expressed a deep sense of loss, both physically and emotionally, which affected their confidence and instilled a pervasive fear in their daily lives.

Furthermore, the study participants expressed that the physical changes stemming from breast cancer and treatment had a substantial impact on their physical well-being and self-perception. According to the participant’s report, their personalities were largely affected by the physical changes they experienced, particularly the loss of one or both breasts due to surgical treatment. This finding aligns with other study findings in which women used quite negative statements about their appearances after mastectomy [14, 29]. This is mainly due to the loss of a breast affecting femininity and the symbolic significance of breasts. Thus, the loss resulted in negative thoughts and feelings of sadness among women with breast cancer. The women in this study frequently reported side effects associated with chemotherapy, such as hair loss, skin changes, and loss of appetite. They also experienced other side effects, including crooked nails, dizziness, palpitations, headaches, changes in skin appearance, and hair loss, which affected their well-being and preferences. These findings are consistent with previous research that identified fatigue, alopecia (hair loss), and constipation as common complaints among breast cancer patients. Particularly troublesome symptoms highlighted in the study included nausea, altered taste, and paraesthesia (abnormal sensations) [30, 31]. These similarities in side effects may be attributed to similar treatments used for breast cancer.

The participants in the study expressed that these physical changes had a profound impact on their lives and affected their mental well-being. This finding is consistent with similar findings reported in other studies [25, 32]. This study emphasises the significant influence of physical changes and symptoms related to chemotherapy on the lives of women diagnosed with breast cancer. Studies from different parts of the world also confirms that coping with diagnosis and treatment is a stressful journey and requires lots of adjustment and changes [25]. Participants in this study expressed their struggles with the physical changes resulting from breast loss, leading them to use breast holders or other methods to conceal the loss as a way to cope. They also experienced reluctance to discuss and disclose their condition due to concerns about others’ opinions and reactions, which, in turn, led to social withdrawal and staying at home. This finding is also consistent with other studies that identified similar themes of emotion suppression, self-stigma, and perceived stigma among breast cancer survivors [24, 33]. Another study explored the experiences of women with breast cancer, highlighting their feelings of health insecurity, the profound impact of the illness on their physical and emotional well-being, and its effects on their marital relationships [34–36]. In addition, these women expressed their concerns and experiences regarding cultural discrimination. The study sheds light on the multifaceted challenges faced by women with breast cancer, encompassing not only their medical journey but also the broader social and emotional dimensions of their lives. They grappled with uncertainties about their health, coping with the physical and emotional toll of the disease, and navigating the impact it had on their relationships. Moreover, they shared their encounters with cultural discrimination, indicating the additional burdens they faced due to societal attitudes and biases surrounding breast cancer.

In this study, women diagnosed with breast cancer expressed the various challenges they faced when accessing therapy. Specifically, they emphasised the financial difficulties they encountered in affording medications, laboratory tests, and transportation for follow-up appointments. Similar findings from other studies have also identified financial hardship as a barrier to early diagnosis [37]. It is possible that the impact of the disease itself, which may limit mobility, coupled with the psychological effects on the patient, contributes to these financial challenges. Additionally, participants in the study shared that the stressful environment of the oncology clinic, along with drug shortages, further exacerbated their difficulties, leading to an overwhelming burden that put their economic well-being at risk. The findings of this study is consistence with previous research findings in India, Ghana, and Kashmir [12, 38], which also revealed that a significant portion of breast cancer patients belong to lower-income groups, making it challenging for them to manage the costs of treatment. The participants in the study expressed that the financial burden was a significant obstacle for breast cancer patients, affecting their ability to actively participate in and adhere to essential medical treatments. They shared their experiences of resorting to extreme measures, such as selling household items, to afford the costs of their therapy. Similar situations were reported in Kashmir, where families had to sell land, jewellery, and borrow money from others to cover the expenses of treatment [38]. These findings highlight the substantial financial barriers and sacrifices faced by breast cancer patients in accessing and affording necessary care. The similarity in these experiences can be attributed to the shared socioeconomic circumstances and living conditions within these societies.

However, women found strength and optimism from various sources, including their faith, the support of loved ones, and the reassurance provided by healthcare professionals. Despite the numerous challenges they faced, these individuals discovered solace and resilience through these pillars of support. Maintaining hope plays a crucial role in cancer patients in their battle against the disease. In this study, participants expressed hope for healing, driven by the early detection of the disease and their belief that God would provide a solution. Another study on cancer patients revealed that individuals perceived the future as being beyond their control and reliant on God’s will, connecting their recovery to divine intervention [35]. Hope is essential in all aspects of life, especially for those facing cancer. In a study conducted in China among patients with metastatic breast cancer, maintaining hope emerged as a coping mechanism. Participants emphasised that living and returning to a normal life fuelled their determination to heal and effectively combat cancer [39]. These findings highlight the significance of hope as a vital component in the lives of cancer patients, sustaining their motivation, and fortitude throughout their journey. The findings of this study indicated that women facing breast cancer derive strength and optimism from various sources, including care and support from healthcare providers, family and friends, and faith-based support from religious communities. These findings are consistent with previous research conducted, emphasising the crucial role of social support during treatment [40, 41]. Such support is vital in helping women cope with the challenges of the disease and facilitating their recovery process. Participants in the study reported that healthcare providers play a significant role in maintaining their hope by providing professional medical advice, treatment options, and tailored emotional support. These providers offer valuable guidance on nutrition during therapy and help in managing treatment side effects. Women consistently express positive experiences and satisfaction with the care provided by healthcare professionals in various studies [4, 17]. In addition to healthcare providers, support from family and friends, as well as faith-based communities, plays a vital role in helping patients maintain hope and navigate the challenges of breast cancer. Loved ones offer both emotional and practical assistance, providing reassurance, communication, empathy, and essential support. This finding aligns with previous studies that have emphasised the significance of emotional support from family, survivorship groups, and spirituality/religiosity as crucial factors in a patient’s journey. Among social sources, immediate family members, particularly husbands, are often reported to be the most supportive [41–43]. These pillars of support not only provide solace but also contribute to the resilience of individuals with breast cancer. In other study finding also their presence and assistance are instrumental in maintaining hope and fostering a positive outlook throughout the challenges associated with the disease [44].

## Conclusion

The findings of this study highlighted the diverse and complex experiences of women with breast cancer, emphasising the emotional implications of unexpected diagnoses and challenges accessing treatment. Despite these difficulties, participants maintain hope through care and support from healthcare providers, family and friends, and faith-based communities.

While acknowledging the support provided by healthcare providers, participants expressed the need for improvements in the oncology clinic environment. The suggestions included addressing overcrowding, ensuring hygienic facilities, and providing separate rooms for different types of cancer patients. The availability of medications and laboratory tests also requires attention from the hospital management.

In conclusion, these findings underscore the importance of comprehensive support and care for women with breast cancer. Enhancing the hospital environment, addressing resource shortages, and prioritising patient well-being are crucial steps toward improving the experiences of breast cancer patients in the study area.

## Limitations

This study has several limitations that should be considered. First, the data were collected from a small number of patients in a single university hospital, which may limit the generalizability of our findings to a larger population. Future research should include a larger and more diverse sample of patients from different healthcare institutions to enhance the external validity of the findings. Second, while interviews are widely recognised as a valuable method for obtaining in-depth understanding, this study did not use interviews, which may have provided richer and more nuanced data on the participants’ experiences. Future studies could consider incorporating interviews as part of the data collection process to gather more comprehensive and detailed information on specific issues. Furthermore, the study would benefit from employing a more standardised and detailed measurement procedure to enhance the reliability of the data. This could involve using validated scales or questionnaires to assess specific aspects of the participants’ experiences and challenges during therapy. Lastly, it is important to note that this study focussed exclusively on patients with breast cancer patients. Different types of cancer may have varying effects on individuals’ life experiences and challenges. Future research could explore and compare the experiences of individuals with different types of cancer to gain a more comprehensive understanding of the broader cancer landscape.

Despite these limitations, the findings of this study contribute to the existing literature and represent an ongoing effort to address the research gap and gain insights into the experiences and challenges faced by Ethiopian women living with breast cancer during therapy.

## Data Availability

The datasets used and/or analysed in relation to the current study are available from the corresponding author upon reasonable request.
